# Canadian Undergraduate Perspectives on Medical Assistance in Dying for Mental Illness: Does Psychiatric Illness Type, Age, and Exposure to Information Influence Acceptance of MAiD?

**DOI:** 10.1177/10499091241247835

**Published:** 2024-05-02

**Authors:** Lori Harper, Christina A. Tomaras, Russell A. Powell, John R. Reddon, Erin Hawrelak

**Affiliations:** 1 Department Psychology, 192290 Macewan University Faculty of Arts and Science, Edmonton, AB, Canada; 2 Department Psychology, 170234 University of Alberta Faculty of Arts, Edmonton, AB, Canada; 3 Department of Law, 113486 University of Saskatchewan College of Law, Saskatoon, SK, Canada

**Keywords:** medical assistance in dying, end of life, euthanasia, assisted suicide, mental illness, depression, schizophrenia

## Abstract

**Background and Objectives:**

In 2027, Canadians whose only medical condition is an untreatable mental illness and who otherwise meet all eligibility criteria will be able to request Medical Assistance in Dying (MAiD). This study investigates the attitudes of undergraduate students towards widening the scope of MAiD for physical illness for certain psychiatric conditions. We were interested in understanding if age, information, and type of mental illness influenced undergraduates’ acceptance or rejection of MAiD for mental illness (MAiD-MI).

**Method:**

413 undergraduate students participated in this study which examined the factors that correlate with the acceptance or rejection of MAiD-MI. Four scenarios were presented in which age (older or younger) and illness type (depression or schizophrenia) were manipulated. Demographic questions and measures assessing personality, religion, and attitudes towards euthanasia were administered. Questions assessing participants’ general understanding of MAiD and their life experiences with death and suicide were also asked.

**Results:**

Most of the participants accepted MAiD-MI for both depression and schizophrenia. As hypothesized, support for MAiD-MI was higher for patients with schizophrenia than for depression. Also as hypothesized, support was higher for older patients than for younger patients. Variables such as religion, personality and political affiliation were also associated with acceptance or rejection of MAiD-MI. Finally, consistent with our hypotheses, participants’ understanding of MAiD and experiences with death and suicide was predictive of support for MAiD-MI.

## Introduction

Proposed changes to the Canadian legislation on Medical Assistance in Dying for physical illness (MAiD-PI) will be made in 2027 to include those Canadians whose only medical condition is an untreatable mental illness (MAiD-MI), further adding to this controversial process.^1-3^

MAiD is legal in the Netherlands, Belgium, Luxembourg, and Switzerland for those suffering from an untreatable medical condition as well as an untreatable psychiatric illness.^4-6^ The research investigating MAiD-MI is limited with most studies finding that depression is the most common diagnosis of those requesting MAiD-MI.^4,5,7,8^

There are several challenges concerning MAiD-MI including difficulties in determining an individual’s mental illness as “untreatable,”^9^ capacities of the individual requesting to make such a decision,^1,10,11^ as well as treatment accessibility. There is also some concern that the values and moral judgements of physicians may influence their opinions of patient prognosis and competence.^6^

The acceptance of MAiD-MI by practitioners and lay people alike may also be adversely affected by the stigma of certain mental disorders. For example, schizophrenia, is one of the most stigmatized mental disorders.^12-15^ Depression has been rated the most visible disorder in that most people know someone with depression or have experienced the illness themselves.^16^ Because of certain movements in Canada, such as “Bell Let’s Talk,” depression appears to have become less stigmatized, particularly when compared to individuals with schizophrenia.^17-23^ Nevertheless, some stigma toward depression remains, including the common misconception that individuals with depression can “get over” or “snap out of” their low mood.^24,25^

Age of the individual requesting MAiD appears to influence acceptance or rejection of MAiD-PI with more perceived legitimacy for and an increased prevalence of MAiD-PI for older adults.^26-29^ Hawrelak and colleagues^30^ also found more acceptance for MAiD-PI for older adults with a possible rationale being that participants believe that older people have more autonomy with respect to making life-ending decisions.^31^ Hawrelak and colleagues also examined undergraduates attitudes towards MAiD-MI and found that most students were supportive of MAiD-MI. In contrast, a recent Canadian study also investigating the attitudes of undergraduate students towards MAiD-MI found that less than one-third (28%) of the students agreed/strongly agreed with the statement that MAiD should be offered to people receiving treatment for mental illness. Of note is that 52% were not aware of this upcoming change to the Canadian legislation.^32^

## The Present Study

We administered a survey to examine the attitudes of Canadian undergraduates toward MAiD-MI and investigate possible factors associated with its acceptance or rejection. A recent poll examined Canadian lay persons’ perspectives on MAiD and found that 65% of respondents support access to MAiD-MI (see https://www.dyingwithdignity.ca/media-center/poll-support-for-medically-assisted-dying-in-canada-2/). However, only 4% of the participants were current post-secondary students. Identifying the attitudes of undergraduate students towards widening the scope of MAiD to include mental illness is important for two reasons. This information will help current policy makers have a clearer perception of the attitudes of undergraduate students towards widening the scope of MAiD, which will no doubt influence education programs regarding the use of MAiD-MI and perhaps shed light on potential biases that individuals may have towards schizophrenia and/or depression. In addition, it is important to assess undergraduates’ perceptions of MAiD-MI as they will be future decision-makers and will therefore influence future legislation.

We chose schizophrenia and depression because schizophrenia is more stigmatizing than depression and as such, we hypothesized that participants will be more accepting of MAiD-MI for schizophrenia than depression. We also hypothesized that participants will be more accepting of MAiD-MI for older patients than for younger patients.^26-29,31^ Based on previous findings for MAiD-PI, we additionally predicted that participants’ religious affiliation, personality traits, stigmatizing beliefs, and political leanings would likewise be associated with acceptance or rejection of MAiD-MI,^33-40^ and hypothesized that having more information about MAiD-MI would be associated with increased acceptance of the procedure.

## Method

### Participants

The study was conducted in the Spring and Fall semesters of 2022 and was completed by 507 participants but 94 participants were eliminated due to excessive missing data and/or evidence of nonpurposeful perseverative responding. The resulting sample size was 413. The study was approved by the Research Ethics Board (Approval no. 101997 on April 29, 2022). Participants provided informed consent and received a course credit of 2% in their Introductory Psychology classes for participating. The data was fully anonymized such that students’ responses could not be identified.

### Information

Participants were randomly assigned to an information group or limited information group. The limited information group received information about what MAiD-MI is, the types of MAiD-MI available, and the eligibility criteria in Canada. The information group read the same material as the limited information group but were given additional information from countries where it is already legal (i.e., Netherlands, Belgium, Switzerland, and Luxembourg). Information about schizophrenia and depression was also provided to both groups. This information is presented in the Appendix (Tables A1 and A2, respectively).

### Measures

#### Scenarios

Participants were asked to read four different scenarios, arranged in random order, where a mentally ill person requests MAiD-MI (see Appendix, Table A3). Scenarios were modified from Levin and colleagues.^11^ Age and mental illness were manipulated in the scenarios, allowing for four possible combinations: Young-Schizophrenia (Y-S), Old-Schizophrenia (O-S), Young-Depression (Y-D), and Old-Depression (O-D). After reading each scenario, participants were asked if that individual should be approved for MAiD-MI, with options being ‘yes, ’no,’ or ‘undecided,’ with a text space provided for those who chose ‘undecided’ to expand on why they chose that option.^1^

#### Demographics

Participants completed a questionnaire about age, gender, political affiliation, marital status, current employment, socioeconomic status, ethnicity, and type of degree/program.

#### Personality

A 10-item short form of the Big Five Personality Inventory^41,42^ was used to assess the five standard dimensions of personality (i.e., extraversion, openness-to-experience, conscientiousness, neuroticism, and agreeableness). Participants were asked to select the extent to which they agreed with the statements on a 5-point scale (1 = disagree strongly, 2 = disagree a little, 3 = neither agree nor disagree, 4 = agree a little, 5 = agree strongly).

#### General Beliefs about MAiD, Death, and Suicide

A modified version of the Weiss and Lupkin (2010)^43^ measure was used which asked participants to rate on a 5-point scale (1 = not at all, 5 = very much) the amount of discussion they have had about MAiD with family members, friends, medical providers, or clergy for both an incurable medical condition and mental illness; the amount of exposure they have had to MAiD through reading, viewing and reflecting upon the topic; and their overall knowledge of MAiD. Participants were also asked about their life experiences with end-of-life decisions and death, mental disorders including schizophrenia and major depressive disorder, and suicide.

#### Attitudes Toward Euthanasia

The Euthanasia Attitudes Scale (EAS)^44^ is a 10-item questionnaire that measures individuals’ attitudes toward euthanasia for physical illness. Participants were asked to rate on a 5-point scale (1 = strongly disagree, 2 = disagree, 3 = undecided, 4 = agree, 5 = strongly agree) the degree that they agree with each statement.^45^

#### Religion

The Duke Religious Index (DRI)^46^ is a 5-item questionnaire that measures an individual’s religious involvement on three dimensions. Organized religion involves group or public gatherings, whereas non-organized religious practices focus on personal activities like prayer. Intrinsic religion refers to the level of devotion.^46^ Participants were asked to indicate on a 6-point scale how accurately each statement describes their usual behaviour or belief regarding religious practices. This measure has been validated in college student samples.^47^

#### Political Affiliation

Participants were asked to indicate if they support a particular political party, with the choices consisting of Conservative, Liberal, NDP, or other. A “left leaning” score was then calculated by assigning Conservative a score of 1, Liberal a score of 2, and NDP a score of 3.

#### Stigma

The Mental Illness Stigma Scale^16^ is a 28-item questionnaire, with seven subscales, that measures attitudes of participants toward people with mental illness. The scale was presented once for the stigma of depression and once for the stigma of schizophrenia. The depression version and the schizophrenia version were presented in random order. Participants were asked to indicate the extent to which they agree or disagree with each statement using a 7-point scale (1 = completely disagree to 7 = completely agree).

## Results

A total of 507 participants completed the study.^1^ Of these, 94 participants had excessive missing data and/or evidence of nonpurposeful perseverative responding on the item data for the scales. Their data was therefore excluded from the analysis. Missing item data was estimated within scale by using regression. Imputed values were rounded to the nearest integer in order to conform to the integer coding of the item data. With the sample of 413 participants, 128 identified as men, 269 as women, and 16 as other, including non-binary, agender, gender fluid, transgender, or preferred not to disclose. The mean age of participants was 21 years (*SD* = 4.93 years; range = 17 to 47 years).

**Figure 1.**
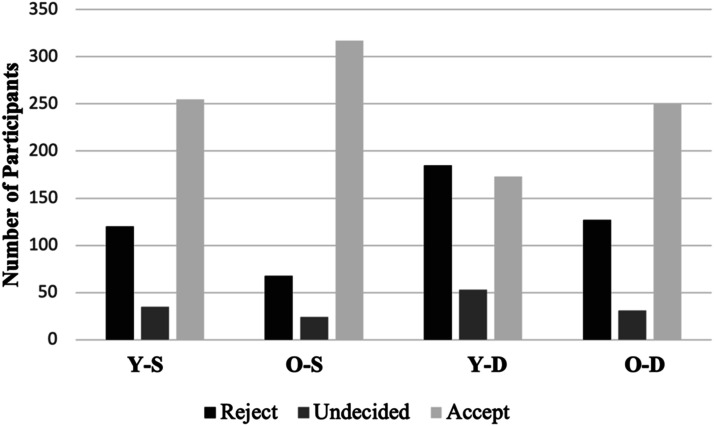
Support for MAiD by participants per Scenario. Scenarios: Y-S: Young-schizophrenia; O-S: Old-schizophrenia; Y-D: Young-depression; O-D: Old-depression.

The number and percentage of participants who rejected, were undecided, or rejected MAiD-MI in each of the scenarios is presented in Table 1 and Figure 1. To assess general level of support for MAiD-MI, a score of 1 was assigned if a participant rejected it, a score of 2 was assigned if they were undecided, and a score of 3 was assigned if they accepted it. These scores were then totaled across the scenarios to create a total acceptance score for each participant. Thus, a total acceptance score of 4 indicated rejection of MAiD-MI in all four scenarios while a score of 12 indicated acceptance in all four scenarios.

**Table 1.** Frequency and Percentage of Participants Who Rejected, Were Undecided, or Accepted MAiD in Each Scenario.

Scenario	Y-S	O-S	Y-D	O-D
Reject	120 (29.3)	68 (16.6)	185 (45.0)	127 (31.1)
Undecided	35 (8.5)	24 (5.9)	53 (12.9)	31 (7.6)
Accept	255 (62.2)	317 (77.5)	173 (42.1)	251 (61.4)
Total	410	409	411	409

Scenarios: Y-S: Young-Schizophrenia; O-S: Old-Schizophrenia; Y-D: Young-Depression; O-D: Old-Depression.

To determine if the patient’s age and type of illness affected level of support for MAiD-MI, a 2 × 2 within-subjects analysis of variance was conducted. The analysis revealed a significant main effect for age, *F*(1, 400) = 41.18, *P* < .0001 (partial *η*^*2*^ = .20), indicating significantly greater acceptance of MAiD-MI for older individuals (*M* = 2.45, *SD* = .83) than for younger individuals (*M* = 2.14, *SD* = .92). We also found a main effect for type of illness, *F*(1, 400) = 43.78, *P* < .001 (partial *η*^*2*^ = .17), with greater acceptance of MAiD-MI for schizophrenia (*M* = 2.46, *SD* = .83) than for depression (*M* = 2.13, *SD* = .92). For both main effects, the effect size was relatively large. We did not find a significant interaction between age and type of illness *F*(1, 400) = 1.77, *P* = .18.

To determine the effect of more information about MAiD-MI, we conducted t-tests on the acceptance scores for participants who received more information vs less information. As shown in Table 2 the effect of more information on greater acceptance of MAiD-MI was significant for total acceptance as well as for the Y-S and O-S scenarios but insignificant for the Y-D and O-D scenarios (*P* < .06). In each case the effect sizes were relatively small.

**Table 2.** Differences in Total Acceptance of MAiD-MI as Well as Acceptance in Each Scenario Between Participants Receiving More Information Versus Less Information About MAiD-MI.

Acceptance of MAiD-MI	More information		Less information		*t*	*df*	*p*	Cohen’s *d*
	*M*	*SD*	*M*	*SD*				
Total	9.59	2.62	8.76	2.81	3.05	399	.002	.305
Y-S	2.46	.84	2.19	.94	3.12	395	.002	.310
O-S	2.69	.68	2.52	.82	2.21	381	.028	.220
Y-D	2.06	.95	1.88	.91	1.93	409	.054	.190
O-D	2.39	.89	2.21	.93	1.89	401	.059	.187

Scenarios: Y-S: Young-Schizophrenia; O-S: Old-Schizophrenia; Y-D: Young-Depression; O-D: Old-Depression.

As shown in Table 3, acceptance scores for MAiD-MI in the different scenarios were positively associated with each other, with the strongest correlations being between the young and old scenarios of each type of illness (Y-S vs O-S: *r* = .573, and Y-D vs O-S: *r* = .559). This table also shows the correlations with the EAS, personality, religion, political affiliation, discussion, exposure and stigma. As shown, EAS scores (which refer to MAiD-PI) were positively associated with acceptance scores in all four scenarios, with stronger correlations for the O-S (*P* < .001) and Y-S (*P* < .01) scenarios than for the O-D and Y-D scenarios (*P* < .05). With respect to personality traits, Conscientiousness was negatively associated with acceptance of MAiD-MI in each scenario except O-S. Nonorganized and intrinsic religion were negatively associated with acceptance across scenarios with exception of an insignificant negative association with nonorganized religion in the O-S scenario. Left-leaning political affiliation was positively associated with acceptance, but only in the depression scenarios. Discussion of MAiD-PI with family was negatively associated with acceptance of MAID-MI in each scenario except O-S. However, discussion of MAiD-MI with family, as well as exposure to MAiD through reflection, were negatively associated with acceptance only in the Y-S scenario. Conversely, experience with someone actively requesting MAiD was positively associated with acceptance across all scenarios except O-S. With respect to stigma, only one subscale—Schizophrenia Visibility, which assesses the extent to which people are bothered by the appearance of those who have schizophrenia—was significantly associated with acceptance of MAiD-MI, and only in the O-S (*r* = −.12) and Y-D (*r* = −.10) scenarios. Total acceptance scores were significantly associated with each of these measures, the strongest correlations being with EAS (r = .174), intrinsic religion (r = .182), and discussion of MAiD-PI with family (r = .178).

**Table 3.** Significant Pearson Correlations With MAiD-MI Acceptance Scores.

Variable		Total	Y-S	O-S	Y-D	O-D
Total acceptance	Total	1				
Scenarios	Y-S	**.795** ^***^	**1**			
	O-S	**.748** ^***^	**.573** ^***^	**1**		
	Y-D	**.788** ^***^	**.498** ^***^	**.357** ^***^	**1**	
	O-D	**.794** ^***^	**.411** ^***^	**.475** ^***^	**.559** ^***^	1
EAS	Score	**.174** ^***^	**.137** ^**^	**.176** ^***^	**.112** ^*^	**.126** ^*^
Personality	Conscientious	**−.150** ^**^	**−.104** ^*^	−.049	**−.175** ^*^	**−.113** ^*^
Religion	Non-organized	**−.151** ^**^	**−.106** ^*^	−.070	**−.104** ^*^	**−.155** ^**^
	Intrinsic	**−.182** ^**^	**−.157** ^**^	**−.099** ^*^	**−.116** ^*^	**−.165** ^**^
Political affiliation	Left leaning	.121*	.041	.042	.128*	.142*
Discussion	MAID-PI with family	**−.178** ^**^	**−.123** ^*^	−.087	**−.143** ^**^	**−.177** ^**^
	MAID-MI with family	**−.098** ^*^	**−.098** ^*^	−.066	−.035	−.071
Exposure to MAiD	Reflection	**−.110** ^*^	**−.137** ^**^	−.062	−.072	−.089
Experience with MAiD	Active euthanasia	**.126** ^*^	**.112** ^*^	.076	**.138** ^**^	.092
Stigma scale for schizophrenia	Visibility	**−.109** ^*^	−.037	**−.120** ^*^	**−.098** ^*^	−.092

Bolded correlations (two-tailed) are significant at the .05 level (*); at the .01 level (**); and at the .001 level (***).

Y-S = Young-Schizophrenia, O-S = Old-Schizophrenia, Y-D = Young-Depressed, O-D = Old-Depressed; EAS = Euthanasia Attitude Scale; MAiD-PI = Medical Assistance in Dying for physical illness; MAiD-MI = Medical Assistance in Dying for mental illness. Because some participants did not respond to some measures, Ns for most variable vary between 398 and 411 (Y-S: 410, O-S: 409; Y-D: 411; O-D: 409). The exception is Left-Leaning political affiliation for which the Ns were around 330 as many participants declined to identify as Conservative, Liberal, or NDP.

## Discussion

### Psychiatric Illness Type Stigma Scales, Euthanasia Attitude Scale, Experience with MAiD

Participants in this study supported MAiD-MI more for schizophrenia than depression and more for older than younger individuals. While this finding may be attributed to the significant stigma and stereotypes associated with schizophrenia as we hypothesized, surprisingly, we found little evidence of an association between stigmatization of schizophrenia or depression and acceptance of MAiD-MI. Only the Visibility item on the modified Stigma in Mental Illness Scale (Schizophrenia Scale)^16^ was significantly associated with acceptance of MAiD-MI and the correlations were relatively weak. Thus, rather than stigma playing a significant role in the greater acceptance of MAiD for schizophrenia, it may simply be that schizophrenia is perceived to be a more serious (i.e., chronic) illness with no hope of recovery and more difficult to live with. Another possibility, however, is that the stigma scale we used is inadequate for assessing the type of stigma involved. Researchers have argued for the automaticity of stereotypes that occur outside of one’s conscious awareness,^48,49^ and it may be that these stereotypes have little effect on participants’ subjective ratings of stigmatizing attitudes toward MAiD-MI.

Data on the reasons why students said “yes” or “no” to MAiD-MI was not gathered and collecting this type of information may be of interest to future researchers. Data for the “uncertain” category was collected and we are hoping to use that data in a different study.

Support for MAiD-MI may have been lower for depression because depression is often viewed as an illness that can improve with treatment and that people with depression can “get over it.” In fact, there is a stereotype that depression is often easily resolved with antidepressants and that depressed individuals can and should shrug off their sadness.^24,25^ More research is needed to sort out the extent to which stigma may influence the acceptance or rejection of MAID-MI.

Participants with higher scores on the Euthanasia Attitude Scale (EAS) tended to support MAiD-MI in all four scenarios. This is not surprising given that higher scores on the EAS suggest higher support for MAiD in general. Also not surprising is that there was more acceptance of MAiD-MI if the participant had experience with someone receiving MAiD-PI which apparently facilitates the acceptability of the assisted dying process.

### Information

The participants who were randomly assigned to the more information group showed significantly greater acceptance of MAiD-MI for the schizophrenia scenarios and marginally significant greater acceptance for the depression scenarios. Although the effect sizes were relatively small, this suggests that information about MAiD-MI can increase the amount of support for it.

### Age of Patient

As expected, the patient’s age in the vignettes was significantly predictive of support for MAiD-MI. These results are consistent with the current literature regarding MAiD-PI.^27,31,37^ It is also consistent with the results of Hawrelak and her colleagues^30^ who found support for both young and old individuals requesting MAiD-MI but with significantly more support for older individuals. Society may view older adults as having greater control over their life decisions,^27,31^ and may also be perceived as having lived a full life which may make ending one’s life less difficult and therefore more acceptable.^50^ Given Canada’s aging population, it is important to augment our understanding of how older adults decide if they want to access MAiD and potential biases in how healthcare professionals determine eligibility for MAiD-MI.

## Religion

Religion was a significant factor in the rejection of MAiD-MI in all four scenarios, as hypothesized, which is not surprising given that many religions condemn suicide. Those who indicated they were involved in either non-organized or intrinsic religions were more likely to reject MAiD-MI. Health professionals’ religious beliefs may result in biased opinions regarding patient prognosis and evaluation of the competency of those seeking MAiD-MI.^6^ Future studies should further investigate the influence of different types of religious beliefs and their subsequent impact on decisions regarding MAiD-MI.

### Political Affiliation

In Canada the NDP is at the left end of the political spectrum, Conservatives are at the right end, and Liberals are in the middle. Political affiliation was significantly associated with accepting MAiD-MI but only in the depression scenarios. This is surprising as other studies have found that being an NDP supporter was a strong predictor of acceptance for MAiD for both terminal and mental illness.^30,40^ In contrast, the Conservative party in Canada has always been opposed to MAiD-PI (see https://www.catholicregister.org/item/32924-federal-conservative-pary-reaffirms-anti-maid-stance) and is also opposed to the expansion of MAiD to include mental illnesses (see CBC https://www.conservative.ca/conservatives-call-on-liberals-to-scrap-expansion-to-assisted-dying-for-vulnerable-canadians-with-mental-illnesses). The present results, however, suggests that the associations may be complex with type of illness playing a significant role in the extent to which party affiliation influences acceptance of MAiD despite the party’s official position on the matter. Future research should explore this possibility.

### Personality

The personality trait of conscientiousness was consistently associated with rejecting MAiD-MI in both the young and older-person depression scenarios and in the young-schizophrenia scenario. Given that people who are conscientious tend to be responsible, diligent, and adhere to norms and rules, it may be that such persons disagree with broadening the scope of the plan to include MAiD-MI. Consistent with this possibility, Parks-Leduc et al.^51^ found that conscientiousness was positively related to ethical decision-making. Thus, having such a trait most likely contributed to viewing MAiD-MI as presumably unethical and hence deterred these participants from accepting MAiD. Data was not collected on the personal values of participants and therefore, it is only speculation what these values were and their impact on ethical decision making. This might be an interesting avenue for future research.

### Limitations

Although helping to provide a foundation for investigating factors that influence the acceptance or rejection of MAiD-MI, the present study has several limitations. In particular, the revised Mental Illness Stigma Scale that we used may not have had the sensitivity to detect certain forms of bias that participants possibly had towards the mental illnesses of schizophrenia or depression. Future studies should explore this using an alternate measure.

## Note

1. The survey materials were revised slightly during the course of the study. After three weeks of data collection, we noticed that a larger number of participants than expected were selecting “undecided” as to whether the person in the scenario should be granted MAiD-MI. This might have stemmed from uncertainty as to whether all possible treatments had been attempted. The scenarios were therefore revised to make clear that all treatment options had been exhausted (see Appendix Table A1). This clarification, however, did not result in a significant change in the proportion of “undecided” responses (O-S: *P* = .67, Y-S: *P* = .82, O-D: *P* = .32, Y-D: *P* = .21) nor in the proportion of “yes” responses (O-S: *P* = .38, Y-S: *P* = .82, O-D: *P* = .84, Y-D: *P* = .20) in each of the four scenarios. On this basis, the decision was made to combine the data from the two versions, resulting in a total of 507 participants (348 who took the original survey and 159 who took the revised survey). After selecting for excessive missing data and/or evidence of perseverative responding, there were 413 participants remaining. The original survey was taken by 287 participants (69.5%) and 126 participants (30.5%) took the revised survey.

## Appendix

**Table A1.** Information Provided to Information Group and Limited Information Group.

Information Group	Medical Assistance in Dying (MAiD) is when a physician or nurse practitioner provides or administers medication that intentionally brings about the death of a qualifying patient who requests it, in compliance with legislation. A physician or nurse practitioner can either directly administer a substance that causes death, such as an injection of a drug (clinician-administered MAiD) or they can provide drugs that the eligible person takes themselves to bring about their own death (self-administered MAiD).
	To be eligible for MAiD in Canada, one must be at least 18 years old and mentally competent, that is, capable of making health care decisions for oneself. One must also request MAiD voluntarily, without external pressure and must give informed consent. The individual must have a grievous and irremediable medical condition with criteria of having a serious illness, disease or disability, be in an advanced state of decline that cannot be reversed, be experiencing unbearable physical or mental suffering from the disease, and the state of decline cannot be relieved under conditions (treatment) that the individual accepts. There are additional safeguards for all requests of MAiD including medical assessment by two independent practitioners, a signed written request for MAiD, independent witnesses to confirm the signing and dating of request, withdrawal of request at any time, and final consent.
	This law is being changed in the near future to include those who have an untreatable mental illness. Canadians whose only medical condition is a mental illness, and who otherwise meet all eligibility criteria, will be eligible for MAiD in March 2023.
	MAiD (also termed euthanasia or EAS) is legal in the Netherlands, Belgium and Luxembourg for those suffering from psychiatric illness. As for the criteria, physicians who approve EAS request must be satisfied that the patient’s request is voluntary and well-considered. The patient’s suffering must be unbearable, with no prospect of improvement, and the physician must have informed the patient about their situation and prognosis. Additionally, there is no reasonable alternative in the patient’s situation, and there must have been a consultation with at least one other independent physician and have exercised due medical care and attention in assisting in patient’s death (Evenbliji et al., 2019). It is likely that such laws in Europe for MAiD, specifically for mental illness, will be similar to the new legislation for Canada (along with current safeguards and criteria) to be in effect March 2023.
	An analysis of 179 reported MAiD cases, in which individuals requested MAiD in Belgium found that 83 of those cases were individuals with a mood disorder such as depression and 22 cases of those with a psychiatric disorder, such as schizophrenia (Dierickx et al., 2017). Moreover, there has been an increase in the absolute number of cases, particularly evident in cases with a mood disorder diagnosis. Despite many treatments for mood disorders, MAiD may be the only option available for those people suffering from severe treatment-resistant depression.
Limited information group	Medical Assistance in Dying (MAiD) is when a physician or nurse practitioner provides or administers medication that intentionally brings about the death of a qualifying patient who requests it, in compliance with legislation. A physician or nurse practitioner can either directly administer a substance that causes death, such as an injection of a drug (clinician-administered MAiD) or they can provide drugs that the eligible person takes themselves to bring about their own death (self-administered MAiD).
	To be eligible for MAiD in Canada, one must be at least 18 years old and mentally competent, that is, capable of making health care decisions for oneself. One must also request MAiD voluntarily, without external pressure and must give informed consent. The individual must have a grievous and irremediable medical condition with criteria of having a serious illness, disease or disability, be in an advanced state of decline that cannot be reversed, be experiencing unbearable physical or mental suffering from the disease, and the state of decline cannot be relieved under conditions (treatment) that the individual accepts. There are additional safeguards for all requests of MAiD including medical assessment by two independent practitioners, a signed written request for MAiD, independent witnesses to confirm the signing and dating of request, withdrawal of request at any time, and final consent.
	This law is being changed in the near future to include MAiD for those who have an untreatable mental illness.

**Table A2.** Symptom Explanation for Depression and Schizophrenia.

Depression Symptom Explanation	Depression is a common and serious mood disorder that can negatively impact one’s health and quality of life, as well as those closest to you. To be diagnosed with depression, symptoms must be present for at least two weeks. Depression isn’t the same as being sad. It’s normal to feel blue or unmotivated from time to time, but depression is more constant. Depression symptoms can vary from mild to severe and can include:
	• Persistent feelings of sadness, hopelessness, worthlessness, and/or emptiness.
	• Loss of interest in activities you once enjoyed.
	• Trouble sleeping or oversleeping.
	• Appetite or weight changes.
	• Fatigue or decreased energy.
	• Difficulty thinking clearly or quickly, remembering details, concentrating, or making decisions.
	• Irritability, frustration, or pessimism.
	• Physical aches and pains including headaches, stomach aches, or neck tension.
	• Recurrent thoughts of death or suicide, with or without a plan to actually do it. (APA, 2021).
Schizophrenia Symptom Explanation	Schizophrenia is a serious mental disorder in which people interpret reality abnormally. Schizophrenia may result in some combination of hallucinations, delusions, and extremely disordered thinking and behaviour that impairs daily functioning and can be disabling. The person is unable to distinguish between real and unreal experiences. People with schizophrenia require lifelong treatment. Some respond well to treatment, but others live a life filled with some of the following symptoms:
	• Positive symptoms: (those “added” to the person) Hallucinations, such as hearing voices or seeing things that do not exist, paranoia and exaggerated or distorted perceptions, beliefs and behaviors.
	• Negative symptoms: (those “taken away” from the person) A loss or a decrease in the ability to initiate plans, speak, express or show emotion or find pleasure.
	• Disorganized symptoms: Confused and disordered thinking and speech, trouble with logical thinking and sometimes bizarre behavior or abnormal movements.
	• Cognition is another area of functioning that is affected in schizophrenia leading to problems with attention, concentration and memory, and to declining educational performance. (APA, 2022).

**Table A3.** Scenarios Provided for Young and Old Schizophrenia and Young and Old Depressed.

Young and Schizophrenia	This individual is 25-years-old and was diagnosed with Schizophrenia five years ago. They are experiencing chronic and severe mental pain and suffering on a daily basis, including having symptoms of vivid hallucinations and delusions. They often think that people are out to hurt them. They also exhibit disorganized thinking and speech, and diminished emotional expression. This individual wishes to request MAiD as there is clear evidence that all treatments, including many different trials of pharmacological medication and numerous sessions of psychotherapy, were unsuccessful. They have also been deemed mentally competent to make such a decision, and they have decided that they do not want to experience any more mental pain.
Old and schizophrenia	This individual is 65-years-old and was diagnosed with schizophrenia five years ago. They are experiencing chronic and severe mental pain and suffering on a daily basis, including having symptoms of vivid hallucinations and delusions. They often think that people are out to hurt them. They also exhibit disorganized thinking and speech, and diminished emotional expression. This individual wishes to request MAiD as there is clear evidence **that all treatments, including many different trials of pharmacological medication and numerous sessions of psychotherapy, were unsuccessful. They have also been deemed mentally competent to make such a decision, and they have decided that** they do not want to experience any more mental pain.
Old and Depressed	This individual is 65-years-old and was diagnosed with Major Depressive Disorder five years ago. They are experiencing chronic and severe mental pain and suffering on a daily basis, including symptoms of diminished mood and lack of pleasure in many activities. They also have feelings of worthlessness and recurrent thoughts of death, extreme fatigue and has difficulty with attention and concentration. This individual wishes to request MAiD as there is clear evidence **that all treatments, including many different trials of pharmacological medication and numerous sessions of psychotherapy, were unsuccessful. They have also been deemed mentally competent to make such a decision, and they have decided that** they do not want to experience any more mental pain.
Young and Depressed	This individual is 25-years-old and was diagnosed with Major Depressive Disorder five years ago. They are experiencing chronic and severe mental pain and suffering on a daily basis, including symptoms of diminished mood and pleasure in many activities. They also have feelings of worthlessness and recurrent thoughts of death, extreme fatigue and difficulty with attention and concentration. This individual wishes to request MAiD as there is clear evidence **that all treatments, including many different trials of pharmacological medication and numerous sessions of psychotherapy, were unsuccessful. They have also been deemed mentally competent to make such a decision, and they have decided that** they do not want to experience any more mental pain.

Note. As noted in the footnote, the original scenarios differed from the revised scenarios. The revised scenarios are present in Table 3. Specifically, the revised sentence regarding treatment exhaustion is indicated by the bolded lines. The original scenarios instead stated: “This individual wishes to request MAiD as there is clear evidence of unsuccessful treatment and they do not want to experience any more mental pain.”
